# Venous thromboembolism in the elderly: efficacy and safety of non-VKA oral anticoagulants

**DOI:** 10.1186/1477-9560-12-21

**Published:** 2014-10-13

**Authors:** Vincent Geldhof, Christophe Vandenbriele, Peter Verhamme, Thomas Vanassche

**Affiliations:** 1 Division of Internal Medicine, University Hospitals Leuven, Herestraat 49, Leuven B-3000, Belgium; 2 Vascular Medicine and Haemostasis, Division of Cardiovascular Diseases, University Hospitals Leuven, Herestraat 49, Leuven B-3000, Belgium; 3 Center for Molecular and Vascular Biology (CMVB), Department of Cardiovascular Sciences, University of Leuven, Herestraat 49, Leuven B3000, Belgium

**Keywords:** Venous thromboembolism, Elderly, Renal insufficiency, Novel oral anticoagulants, Non-VKA-acting oral anticoagulants

## Abstract

Increasing age and renal impairment are risk factors for venous thrombosis but also for anticoagulant-induced bleeding. In large-scale phase III trials, non-VKA oral anticoagulants (NOACs) were at least as effective and safe for the treatment of acute venous thromboembolism as warfarin. Here, we review the efficacy and safety of dabigatran, rivaroxaban, apixaban and edoxaban in the subgroups of elderly patients (≥75 years) and patients with impaired renal function (creatinine clearance ≤50 ml/min). In all phase III trials, the efficacy of NOACs in the prevention of recurrent VTE was conserved both in the elderly subgroup and in the subgroup with impaired renal function. In a meta-analysis of the pooled results, NOACs reduced VTE recurrence compared with warfarin in elderly patients. In elderly patients and patients with impaired renal function, the safety of NOACs was in line with the results of the overall study.

NOACs may offer an effective, safer and more convenient alternative for VKAs also in the elderly. However, the efficacy/safety profile of NOACs in the aged population needs to be confirmed in real-life.

## Introduction

Venous thromboembolism (VTE), with an annual incidence of 1.5-3.0 cases per 1000 individuals, is the third most common cause of cardiovascular disease and death after myocardial infarction and stroke [1-3]. VTE constitutes a spectrum ranging from asymptomatic distal deep venous thrombosis (DVT) and subsegmental pulmonary embolism (PE), to limb threatening DVT and fatal PE [2].

Age is an important risk factor for VTE and VTE recurrence. The risks for DVT and PE are 4 to 6 times higher in patients above 70 years old compared to younger patients [4,5], and this risk doubles with each decade of aging [6]. Importantly, elderly patients also exhibit a higher case-fatality rate due to more frequent fatal PE (odds ratio in patients aged >75 years 2.31 [7]) and coexistent comorbidities. The diagnosis of VTE in the elderly also poses particular challenges, as an atypical presentation and a reduced sensitivity and specificity of both clinical scoring systems and laboratory parameters may impede a timely diagnosis [8].

Age is not only a risk factor for VTE, but also increases the risk of bleeding related to anticoagulant treatment. The risk for major bleeding complications in anticoagulated patients is 2.5% per year in those aged over 80, compared to 0.9% per year in younger patients [8-11]. Until recently, Vitamin K Antagonists (VKAs) were the only available oral anticoagulants. Due to their unpredictable pharmacokinetic profile, the management of VKAs can be challenging. INR variability predisposes to both reduced efficacy and increased bleeding risk. This is particularly problematic in the elderly, where comorbidities and concomitant medication may further increase the risk of bleeding [12].

Altogether, these limitations and complications of VKA therapy may dissuade physicians from prescribing VKAs to the elderly [8,13]. Low Molecular Weight Heparins (LMWHs) provide a more predictable anticoagulant effect but require parenteral administration, limiting their long-term use [8].

Over the recent years, non-VKA-acting Oral Anticoagulants (NOACs) have become available for the prevention and treatment of VTE and for the prevention of atrial fibrillation related stroke. The efficacy and safety of the thrombin inhibitor dabigatran etexilate and the factor Xa inhibitors rivaroxaban, apixaban and edoxaban for the treatment and secondary prevention of VTE have been demonstrated in large phase III programs [1,3,14-18].

NOACs offer several advantages over VKAs. First, due to their more predictable pharmacodynamics and limited drug interactions, NOACs do not require routine coagulation monitoring. Secondly, they exhibit a faster on- and off-set compared to VKAs, which may offer a benefit when bleeding complications occur, and which facilitates the periprocedural management of anticoagulation.

In elderly patients, however, these characteristics of NOACs may be challenging. Although the use of a fixed dose regimen is convenient both for patients and physicians, there could be a risk for increased drug exposure in patients with reduced drug clearance [8]. This issue is of particular importance for the direct thrombin inhibitor dabigatran etexilate, which is mainly eliminated via the kidney.

Furthermore, the shorter half-life of NOACs poses a potential risk when doses are skipped due to non-compliance or forgetfulness. It is uncertain if this variability may unfavorably alter the balance between risk and benefits of real-life treatment in the elderly patients [19].

The purpose of this study was to review the efficacy and safety of NOACs in the elderly and in renal impaired patients.

## Methods

### Search strategy

We limited this review to NOACs that are either approved for the treatment of VTE or under consideration for such approval by the European Medicine Agency. All phase III randomized controlled trials comparing the selected NOACs with VKA therapy for the initial treatment of VTE were included. From these trials, all available analyses for age and renal function subgroups were obtained from the original publications, from their online supplements, or abstract presentations at major conferences. We compared patients ≥75 years with patients <75 years, and patients with impaired renal function with patients with preserved renal function, using a cutoff of a creatinine clearance (CrCl) of 50 ml/min (Cockroft-Gault formula), exept in the RECOVER subgroups were the reported cutoff for age was >75 *vs*. ≤75 and for renal function ≥50 ml/min *vs*. <50 ml/min.

For each trial, risk ratios (RRs) and 95% confidence intervals (95% CIs) of NOACs *vs*. VKA therapy were obtained or calculated if not reported. For subgroups, P interaction was calculated [20]. We compared recurrence rates across trials and in a pooled analysis for patients over 75 years old *vs*. younger patients, and for patients with a reduced *vs*. normal renal function for NOAC treatment and VKA treatment arms separately. Furthermore, we performed a meta-analysis for the relative efficacy as well as safety of NOACs *vs*. VKA in patients over 75 years and in patients with a reduced renal clearance. RRs and corresponding 95% CIs were calculated for the individual trials and pooled according to the Mantel-Haenszel method, using a random effect model (Review Manager 5.2).

### Study outcomes and definitions

In all selected trials, efficacy outcomes were recurrent VTE or death related to VTE. The primary safety outcome was major bleeding in all trials besides the HOKUSAI-VTE trial, where the combination of major and clinically relevant non-major (CRNM) bleeding was the primary safety outcome. The definition of major bleeding was similar in all included studies and conform the International Society on Thrombosis and Haemostasis guidelines [21].

## Results

**Figure 1:**
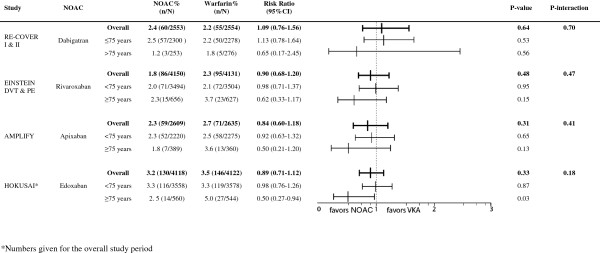
Efficacy according to age.

**Figure 2:**
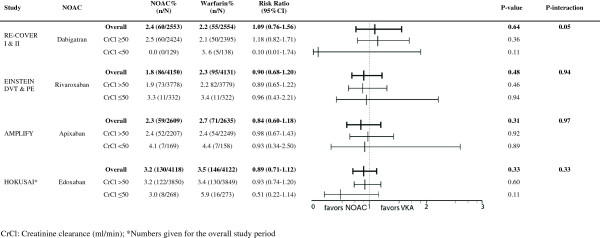
Efficacy according to renal function.

**Figure 3:**
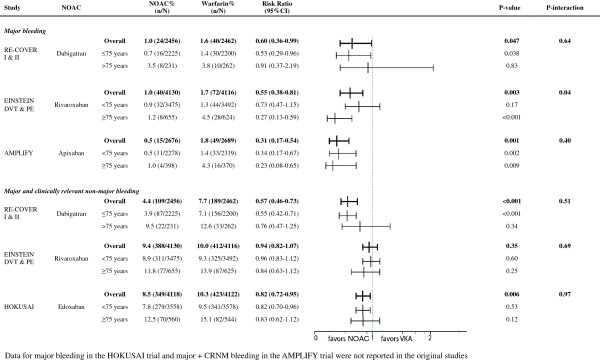
Safety according to age.

**Figure 4:**
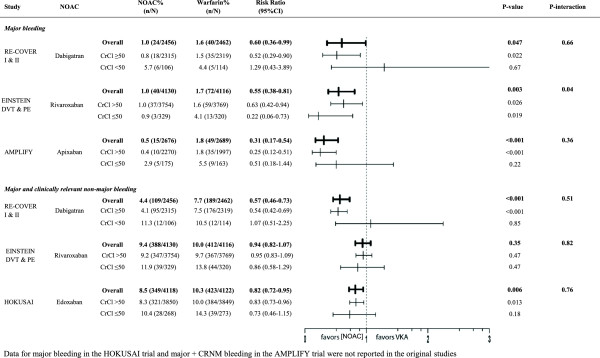
Safety according to renal function.

Four NOACs were either recently approved (rivaroxaban, dabigatran) or are in the regulatory approval process (apixaban, edoxaban) for treatment of VTE. The study characteristics are summarized in Table 1. The different subgroup analyses for efficacy and safety outcomes in the elderly and renally impaired are summarized in Figures 1, 2, 3, and 4.

**Table 1:** **RCT’s of NOACs *vs*. Warfarin in acute therapy of VTE**

**Author, year**	**Trial name**	**Drug and dose**	**Initial regimen**	**N pts, overall**	**N pts ≥75 years (%)**	**N pts CrCl ≤50 ml/min (%)**
*Schulman*, *2009 Schulman*, *2014*	**RE-COVER I-II**	Dabigatran 150 mg bd	Heparin lead-in	5107	259 (12)	167 (5.2)
*Bauersachs*, *2010 Buller*, *2012*	**EINSTEIN DVT-PE**	Rivaroxaban 20 mg od	Rivaroxaban 15 mg bd for 3 weeks	8281	1283 (18)	664 (8.0)
*Agnelli*, *2013*	**AMPLIFY**	Apixaban 5 mg bd	Apixaban 10 mg bd for 1 week	5395	768 (14)	327 (6.2)
*Buller*, *2013*	**HOKUSAI**	Edoxaban 60 or 30* mg od	Heparin lead-in	8292	1004 (12)	541 (6.6)

RCT, randomized controlled trial; bd, bis in die; od, omne in die. RE-COVER I-II [17,18,22]; EINSTEIN [14-16,19,23]; AMPLIFY [1]; HOKUSAI [3] - *30 mg od in patients with CrCl 30-50 ml/min, body weight <60 kg or concommitant use of strong Pgp inhibitors.

### Dabigatran

In RE-COVER I and II, only 10.4% (N = 529) were aged ≥75 years and only 5.2% (N = 267) had a CrCl ≤50 ml/min [17,18]. There was no significant difference in efficacy of heparin followed by dabigatran 150 mg BID *vs*. heparin/VKA in patients aged over 75, however, only 8 events occurred in this age group, limiting statistical power (Figure 1). In the subgroup of patients with reduced renal function, the rate of recurrence was lower in patients randomized to dabigatran compared to VKA-treated patients, however, this was non-significant due to the large confidence interval (RR 0.11 95% CI 0.01-1.88) (Figure 2).

Both elderly patients and patients with reduced renal clearance had a markedly higher bleeding risk regardless of their treatment with dabigatran 150 mg BID or warfarin Figures 3 and 4). Although dabigatran was associated with a reduction in major bleeding in the overall trial results [17,18], this reduction was no longer seen in the subgroups of elderly patients and patients with reduced renal clearance, where the rate of major bleeding was similar in patients treated with dabigatran and warfarin with a RR of 0.91 (95% CI 0.37-2.19) for age >75 and 1.29 (95% CI 0.43-3.89) for clearance <50 ml/min. (Figures 3 and 4) [24,25]. Importantly, the reported subgroup analyses excluded the heparin lead-in phase, which restricts the evaluation of the safety of the overall strategy.

### Rivaroxaban

Together, both EINSTEIN trials included 1283 patients (18%) aged ≥75 [14,15], and 664 patients (8%) with renal impairment. The risk for recurrence increased from 2.1% in younger patients to 3.7% in patients over 75 years old (P = 0.02) in patients randomized to warfarin (Figure 1). In contrast, there was no age-dependent increase in recurrence in the rivaroxaban-arm: 2.3% in aged ≥75 *vs*. 2.0% in aged <75 years (p = 0.07). In patients with reduced renal function, the rate of recurrence was increased by over 50% in both rivaroxaban- and VKA-treated patients. There was no significant effect of renal function on the relative efficacy of rivaroxaban compared to VKA (Figure 2).

**Figure 5:**
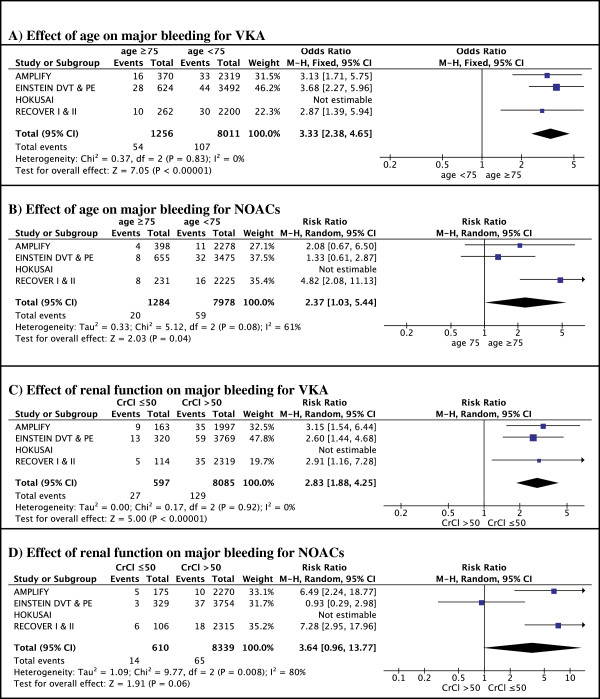
Effect of age and renal function on major bleeding for NOACs and VKA.

In the EINSTEIN trials, treatment with rivaroxaban was associated with a 45% overall reduction in major bleeding [16]. When analyzed by subgroup, the reduction in major bleeding was more prominent in the age group ≥75 years (RR 0.27, 95% CI 0.13-0.59; p = 0.001) (Figure 3) [16,19]. In warfarin-treated patients, major bleeding rates were 3.5 times higher in patients over 75 years old (4.5 *vs*. 1.3%, p = 0.02). In contrast, age above 75 did not significantly increase the bleeding rates in rivaroxaban-treated patients (1.2 *vs*. 0.9%, p = 0.62) (Figure 5). The reduction in major bleeding with rivaroxaban *vs*. warfarin was consistent in patients with conserved and patients with impaired renal function (RR 0.63 (95% CI 0.42-0.94) and 0.22 (95% CI 0.06-0.78), respectively) (Figure 4).

### Apixaban

In AMPLIFY, 14% (N = 768) of the patients were aged 75 years or more and 6.2% (N = 327) had a reduced renal function [1]. Compared to those aged <75, there was a trend towards a better efficacy of apixaban compared with warfarin in older patients (Figure 1). In patients treated with warfarin, there was a 44% increase in event rates in older patients compared to younger patients. In contrast, in the apixaban-arm, event rates in older patients were not elevated compared to younger patients (Figure 1). Subgroup analysis for renal function showed similar efficacy in both renal function classes (Figure 2). However, either type of anticoagulant treatment was less effective in renally impaired patients, as shown by a 70% increase in recurrence rates compared to patients with normal renal function, regardless of the treatment (Figure 2).

With regard to the primary safety outcome, apixaban significantly reduced major bleeding with 70% compared to warfarin in the overall analysis, and this reduction remained significant both in younger and in elderly patients (Figure 3). The RR in patients with normal renal function and reduced renal function was similar (P interaction 0.36) but the safety benefit was no longer significant in patients with impaired renal function (P-value 0.22) (Figure 4) [1].

### Edoxaban

In the HOKUSAI trial 12% (N = 1004) of included patients were aged 75 years or older, and 6.6% (N = 541) had a CrlCl ≤50 ml/min [3]. Subgroup analysis showed a statistically significant 50% reduction of recurrent VTE in edoxaban-treated patients compared to VKA-treated patients for those patients aged 75 years or older (RR 0.50; 95% CI 0.26 to 0.95, p = 0.03) whereas there was no apparent difference in younger patients (RR 0.98 (95% CI 0.76-1.26; p = 0.87) (Figure 1). Edoxaban was equivalent to warfarin in terms of bleeding outcomes, irrespective of the age subgroups (Figure 3). The HOKUSAI trial was unique for the fact that a planned dose reduction (30 mg instead of 60 mg) was foreseen for patients with CrCl 30–50 ml/min or a body weight less than 60 kg. In patients with reduced renal function, there was a non-significant trend towards greater efficacy of edoxaban *vs*. warfarin (RR of 0.51; 95% CI 0.22-1.17) compared to patients with a normal renal function (RR of 0.93; 95% CI 0.74-1.20) (Figure 2). Bleeding risk in this subgroup was similar with edoxaban and warfarin (Figure 4).

### Pooled data and meta-analysis

**Figure 6:**
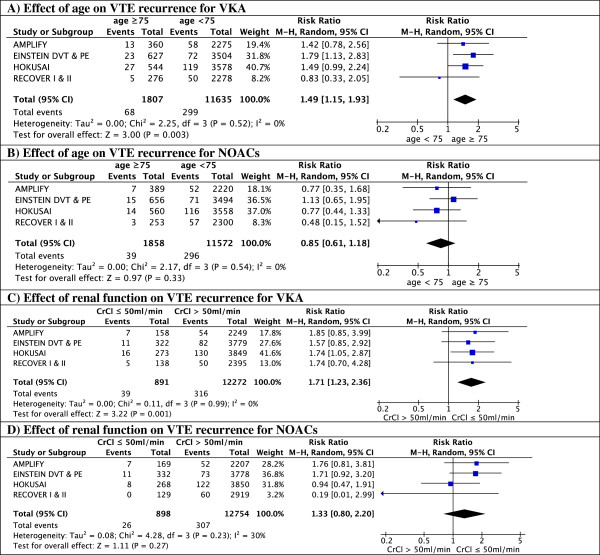
Effect of age and renal function on recurrence of VTE for NOACs and VKA.

**Figure 7:**
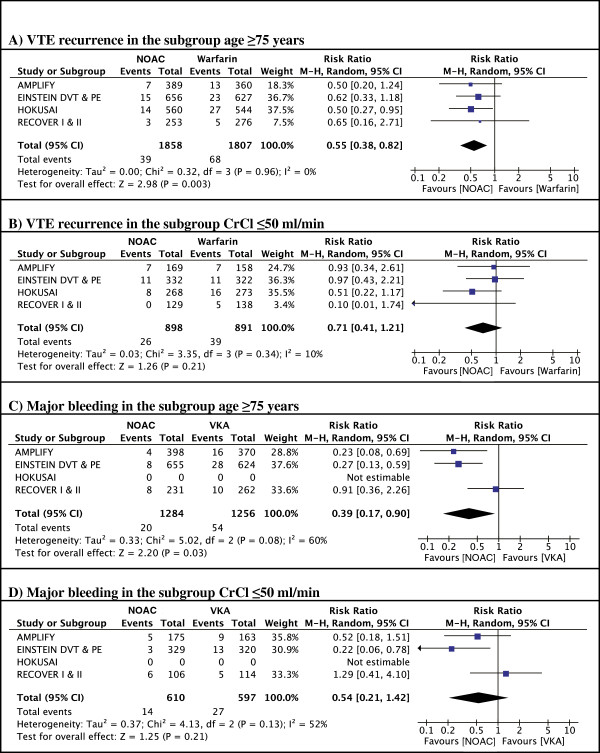
Meta-analyses.

In patients treated with warfarin, the rate of VTE recurrence was higher in patients aged over 75, with a pooled risk ratio of 1.47 (95% CI 1.13-1.90) compared to warfarin-treated patients <75 years. In contrast, patients ≥75 treated with NOACs did not have an increased recurrence compared to younger patients (RR 0.83, 95% CI 0.59-1.15) (Figure 6A, B). Thus, in a meta-analysis of all patients over 75, NOACs were more effective compared to warfarin (risk ratio for recurrence 0.55 (95% CI 0.38-0.82) (Figure 7A).Renal impairment was associated with an increased risk of recurrence in the warfarin-arms of all trials. Although not significant in most individual trials, pooled data show a significant 71% risk increase in patients with reduced renal clearance. A similar increase in recurrence was noted in patients with reduced renal function treated with apixaban or rivaroxaban, but not in patients treated with dabigatran or edoxaban. A pooled analysis showed no significant difference in relative efficacy between NOACs and warfarin in patients with renal dysfunction (relative risk ratio 0.71, 95% CI 0.41-1.21) (Figure 7B).

Because the rates of major bleedings in the subgroups of interest were not reported for the HOKUSAI trial, the edoxaban-study was not included in the safety meta-analyses. Overall, NOACs were associated with a reduced risk of major bleeding in elderly patients compared to warfarin (relative risk ratio 0.39, 95% CI 0.17-0.90) (Figure 7C). Elderly patients treated with apixaban or rivaroxaban appeared to have a greater safety benefit compared to VKAs than those treated with dabigatran (relative risk ratios 0.23, 95% CI 0.08-0.69 and 0.27, 95% CI 0.13-0.59 for apixaban and rivaroxaban respectively *vs*. 0.91 95% CI 0.36-2.26 for dabigatran). In the subgroup of patients with reduced renal function, the pooled analysis shows a trend towards a reduction in bleeding risk (relative risk ratio 0.54, 95% CI 0.21-1.42) (Figure 7D).

## Discussion

We reviewed the available data regarding efficacy and safety in the phase III trials of NOACs for the acute treatment of VTE in the elderly and renally impaired. The pooled data presented here give information on two clinically relevant questions: first, what is the impact of age or reduced renal clearance on the risk of recurrence or bleeding, and secondly, how do NOACs compare to VKA in these subgroups.

In the subgroup of elderly patients or patients with reduced clearance, treatment with VKA is both less effective (50 to 70% increase in recurrence) and less safe (about 3-fold increased risk of bleeding). This is in line with finding from previous studies, reporting an increased risk for both bleeding and (recurrent) thrombosis in these frail patients when treated with conventional anticoagulation [4,6,26].

This could be the result of a reduced quality of VKA management in frailer patients, or an increased susceptibility for thrombotic or bleeding complications. To our knowledge, time into therapeutic range (TTR) as a function of age or renal function subgroups has not been reported for these trials.

In contrast with VKA, NOACs were not associated with a significant increase in recurrence in subgroups of frail patients. In the meta-analyses, NOACs were more effective than warfarin in the subgroup of elderly patients, and there was a trend towards better efficacy in patients with reduced renal function. There are two possible explanations for this improved efficacy, both of which likely contribute to the observed effect.

First, the more predictable pharmacokinetics of NOACs may offer better protection against recurrence than warfarin in subgroups with more challenging warfarin management [12]. If the improved efficacy of NOACs *vs*. warfarin in these subgroups is due to more stable pharmacokinetics, NOACs could also be expected to be associated with a lower risk of bleeding in these subgroups. This was true for apixaban and rivaroxaban, but not for dabigatran.

Secondly, accumulation of NOACs due to reduced renal clearance may lead to a higher intensity of anticoagulation, which may in turn overcome an increased prothrombotic state in these subgroups. This is supported by the finding that dabigatran, which is approximately 80% renally cleared, was associated with the strongest reduction in recurrence in patients with renal insufficiency.

Altogether, the data from the NOAC trials in patients with VTE suggest that NOACs offer better overall safety and efficacy profiles in elderly patients, and comparable safety and efficacy profiles in patients with moderate renal insufficiency. Although the overall findings are reassuring with regards to the use of NOACs in these subgroups of frail patients, some points should be highlighted.

First, interpretations regarding subgroup analyses should always be made with caution, since those trials are insufficiently powered for subgroups. In a meta-analysis comparing NOACs *vs*. warfarin in atrial fibrillation [27], which had considerably more statistical power due to the larger trial size and the higher mean age of included patients, NOACs were associated with a trend towards better efficacy and comparable safety in the elderly and renally impaired. Despite the different patient population, it is reassuring that the global results are in line with our findings. Second, combined results for elderly patients with renal dysfunction are not reported, hence we cannot provide data on this specific group. Third, it should be noted that, although NOACs performed as good as VKA in these patients, the absolute rates for both recurrence and (major) bleeding were higher in patients with moderately reduced renal function (up to 7-fold increase). This stresses the importance of renal function as a risk factor, and posed a dilemma for the clinician as a dose-reduction in patients with a reduced renal function might not be the best strategy to improve the overall risk-benefit ratio. Interesting in this regard are the findings from the HOKUSAI trial. Although the HOKUSAI study suggests that the use of a prespecified dose reduction ensured a similar safety as well as a conserved efficacy in patients with reduced renal function compared to normal renal function, these results were not different from the other trials, where no such dose reduction was prespecified. Lastly, all trials used different types and duration of initial therapies, and not all bleeding outcomes in relation to the subgroups of interests have yet been reported, potentially confounding the safety profile in our meta-analysis.

Perhaps the most challenging question is to which extent these results from clinical trials apply to real-world patients. In general, clinical trial patients represent a selected population with minimal comorbidities and concomitant medication use and optimal compliance, all of which are particular challenges in elderly patients. Therefore, the discrepancy between clinical trial populations and the ‘real world’ patients is likely to be even larger in older age groups. Furthermore, the trials included very few patients over 80 or 90 years old, and excluded patients with severe renal insufficiency or with a high risk of bleeding. Thus, the frailest patients may not be adequately represented. Nonetheless, the same phenomenon applies to VKA treatment. The quality of warfarin management, reflected in the TTR, ranged from 57% in EINSTEIN DVT [14] to 64% in HOKUSAI [3] compared to about only 40% in real life registries and daily clinical practice [3]. Although TTR was not reported as a function of age subgroups, the quality of VKA strengthens the external validity of these studies [28].

These limitations highlight the importance of post-marketing registries in real-world patients [19]. In a recent Food and Drug Administration (FDA) report of bleeding complications of dabigatran *vs*. warfarin in atrial fibrillation patients derived from insurance-claim and administrative data, there was no evidence that real-world rates of bleeding with dabigatran were higher than with warfarin [29]. This report provides some, albeit indirect, reassurance about the external validity of the trial results, but more real-world data is needed in the appropriate patient group.

The subgroup data presented here have important clinical implications. The available evidence demonstrates that NOACs are at least as safe as VKA treatment, and may offer a better efficacy in elderly patients or patients with moderate renal dysfunction who otherwise match the in- and exclusion criteria of the clinical trials. In the absence of head-to-head comparisons between NOACs, it is not possible to directly compare different NOACs. Nevertheless, the relative efficacy of NOACs in the elderly and in patients with low renal clearance was very similar.

In conclusion, data from the published trials suggest that NOACs may be more effective than warfarin in the treatment of acute VTE in the elderly population, and as effective as warfarin in patients with moderate renal dysfunction. Furthermore, there was no increased risk of bleeding with NOACs compared to warfarin in these subgroups. However, these results are based on a relatively small numbers of events, highlighting the need for appropriately designed and powered studies in these subgroups. Furthermore, the question remains to which extent these results can be translated to a less selected population. Future data from observational studies and registries are needed to clarify the benefit/risks profile of NOACs in the real world.

## Competing interests

Peter Verhamme has received research grants or served as a consultant or speaker for: Bayer HealthCare; LeoPharma; Boehringer Ingelheim; Daiichi Sankyo; Pfizer; Sanofi-Aventis, ThromboGenics.

The other authors declare that they have no competing interests.

## Authors’ contributions

VG performed literature search, VG and TV performed statistical analysis, all authors contributed to writing of the manuscript. All authors read and approved the final manuscript.
